# Dominance of phage particles carrying antibiotic resistance genes in the viromes of retail food sources

**DOI:** 10.1038/s41396-022-01338-0

**Published:** 2022-10-26

**Authors:** Pedro Blanco-Picazo, Sara Morales-Cortes, María Dolores Ramos-Barbero, Cristina García-Aljaro, Lorena Rodríguez-Rubio, Maite Muniesa

**Affiliations:** 1 Department de Genètica, Microbiologia i Estadística, Universitat de Barcelona, Diagonal 643. Edificio Prevosti. Planta 0, E-08028 Barcelona, Spain; 2 Departmento de Fisiologia, Genética y Microbiología, Universidad de Alicante (UA), 03080 Alicante, Spain

**Keywords:** Bacteriophages, Antibiotics

## Abstract

The growth of antibiotic resistance has stimulated interest in understanding the mechanisms by which antibiotic resistance genes (ARG) are mobilized. Among them, studies analyzing the presence of ARGs in the viral fraction of environmental, food and human samples, and reporting bacteriophages as vehicles of ARG transmission, have been the focus of increasing research. However, it has been argued that in these studies the abundance of phages carrying ARGs has been overestimated due to experimental contamination with non-packaged bacterial DNA or other elements such as outer membrane vesicles (OMVs). This study aims to shed light on the extent to which phages, OMVs or contaminating non-packaged DNA contribute as carriers of ARGs in the viromes. The viral fractions of three types of food (chicken, fish, and mussels) were selected as sources of ARG-carrying phage particles, whose ability to infect and propagate in an *Escherichia coli* host was confirmed after isolation. The ARG-containing fraction was further purified by CsCl density gradient centrifugation and, after removal of DNA outside the capsids, ARGs inside the particles were confirmed. The purified fraction was stained with SYBR Gold, which allowed the visualization of phage capsids attached to and infecting *E. coli* cells. Phages with *Myoviridae* and *Siphoviridae* morphology were observed by electron microscopy. The proteins in the purified fraction belonged predominantly to phages (71.8% in fish, 52.9% in mussels, 78.7% in chicken sample 1, and 64.1% in chicken sample 2), mainly corresponding to tail, capsid, and other structural proteins, whereas membrane proteins, expected to be abundant if OMVs were present, accounted for only 3.8–21.4% of the protein content. The predominance of phage particles in the viromes supports the reliability of the protocols used in this study and in recent findings on the abundance of ARG-carrying phage particles.

## Introduction

Horizontal gene transfer is a key mechanism by which bacterial populations acquire genetic diversity. Understanding the mechanisms underlying the mobilization of antibiotic resistance genes (ARGs) is of paramount importance because of their involvement in the emergence of resistant and multi-resistant clones. The substantial increase and spread of antibiotic resistances have rendered many infections untreatable by antimicrobials and is recognized by the World Health Organization (WHO) as one of the most important health problems of the XXI century [1].

Several mechanisms of antibiotic resistance transfer have been described to date [2, 3], the most canonical being transformation with free DNA, transduction by bacteriophages, and conjugation involving plasmids and integrative conjugative elements. Transformation, the natural incorporation of extracellular DNA by cells, has been poorly investigated [4]. In contrast, numerous studies have focused on conjugation, in which ARG transfer occurs between related cells by means of a conjugative pilus. There is plenty of evidence for ARG location in plasmids, with many encoding several ARGs, and ARG mobilization by conjugation can be readily demonstrated experimentally. Consequently, the prevailing view is that conjugation is the most frequent mechanism of ARG transfer, particularly in highly dense microbiomes such as the gut [3].

Although transduction, first described many decades ago, is well-established as a strategy of ARG mobilization [3], its impact has been generally considered far below that of conjugation. However, the high frequency with which phages carry ARGs, revealed in a growing number of studies, suggests the role of this mechanism has been underestimated. This mounting evidence, together with recent metagenomic data indicating that phages are the most abundant viruses in existence and present in all viromes, has stimulated new interest in phages as vehicles of ARG transfer.

However, the sequencing of complete phage genomes carrying ARGs and experimental evidence for ARG transduction by phages is scarce compared to the number of studies simply confirming the presence of ARGs in the phage DNA fraction. A possible explanation is that ARG-containing phage particles may be generated by generalized or lateral transduction mechanisms [5, 6], resulting in particles that only package a large fragment of bacterial DNA [7].

The impossibility of propagating and isolating pure suspensions of transducing phage particles in high titers means experimental ARG transduction is extremely difficult. In addition, as transducing particles only contain bacterial DNA, they cannot be identified by sequencing. Due to these limitations, it has been suggested that studies analyzing the phage DNA fraction have overestimated the frequency of ARGs and other bacterial genes, due to the contaminating effect of non-packaged DNA or ARGs in outer membrane vesicles (OMVs). Yet the assumption that 16 S rRNA genes in the phage fraction necessarily arise from DNA contamination is also questionable, as 16 S rRNA genes can be packaged in phage particles in the same way as any other part of the bacterial genome [8, 9]

Using protocols developed to eliminate the possibility of erroneously detecting ARGs other than those inside transducing phage particles [10–14], here we present additional evidence that, in those studies focusing on the viral fraction of different samples, phage particles are major carriers of ARGs.

## Materials and methods

### Samples

Two samples of minced chicken meat (“Chicken 1” and “Chicken 2”), a pool of two samples of mussels (“Mussels”), and a pool of three samples of Mediterranean whiting (“Fish”) were used in this study. These sample types were selected because they were previously found to contain phage particles carrying ARGs [10, 15]. All samples were purchased from local supermarkets in the area of Barcelona (Spain) during 2020–2022. All of them were fresh samples as they had not undergone any packaging or freezing process and were analyzed within 24 h after purchase.

Twenty g of each pool of samples were homogenized for 2 min in 60 ml of phage buffer (100 mM NaCl, 10 mM MgCl_2_, 50 mM Tris-HCl, 0.01% gelatin, pH 7.5) with the Stomacher homogenizer (IUL Instruments GmbH, Königswinter, Germany). Stomacher bags with filters (Afora, Barcelona, Spain) were used to enhance the separation of solid waste from the liquid fraction containing the microorganisms.

### Bacterial and viral indicators

Total aerobic microorganisms and total aerobic microorganisms resistant to ampicillin (amp) in the homogenate were evaluated on tryptone soy agar (TSA) or TSA with amp (100 μg/ml) at 37 °C.

Total *Escherichia coli* and *E. coli* resistant to amp in the homogenates were determined on Chromocult Coliform Agar (Merck, Darmstadt, Germany) or Chromocult Coliform Agar with amp (100 μg/ml), respectively. Incubation was first performed for 2 h at 37 °C to adapt potentially damaged microorganisms and then overnight at 44 °C. Ten percent of the blue colonies presumed to be *E. coli* were confirmed with the indole test and grown in McConkey agar. Each supernatant was analyzed for all the indicators in triplicate.

### Purification of phage particles

Fifty ml of each homogenate was filtered through 0.22 μm low protein binding polyethersulfone (PES) membranes (Millex-GP, Merck Millipore). The filtrates were treated with chloroform (10% v/v) and shaken for 5 min at room temperature. Then, the two-phase mixture was separated by centrifugation at 4000 × g for 10 min. The collected aqueous phases were treated with 100 U of DNAse I (Sigma-Aldrich. St. Louis, MO, US) at 37 °C for 1 h using the reaction buffer provided by the manufacturer. DNase I was then inactivated by heating at 75 °C for 5 min.

### Propagation cultures of ARG-carrying phages

The ability of the ARG-containing phage particles to infect and propagate in enrichment cultures of the *E. coli* WG5 (ATCC 700078) host strain was evaluated. This strain was selected for its high sensitivity to infection with phages of different types [16] and because its genome does not contain prophages or any of the ARGs targeted in this study [17].

The propagation cultures were prepared with 1 ml of each phage suspension after the DNAse treatment and 1 ml of *E. coli* WG5 at the mid-log growth phase (OD_600_ of 0.3) in 8 ml of Luria-Bertani broth (LB) and incubated overnight at 37 °C with shaking. After incubation, phages were purified by filtration through 0.22 µm low protein binding PES membranes and treated with chloroform and DNase, as indicated above.

### Phage particle purification by CsCl density gradient centrifugation

Phage suspensions after propagation were subjected to purification by cesium chloride (CsCl) density gradient centrifugation using Ultra-Clear Thin Wall tubes (Beckman Coulter. Brea, CA, US), 1 ml of 20% sucrose (w/v) and three densities of CsCl (1.3, 1.5, and 1.7 g/ml) [18]. Samples were ultracentrifuged at 22 000 × *g* for 2 h at 4 °C in a Swinging-Bucket SW-41 Rotor in a Beckman ultracentrifuge.

**Fig. 1:**
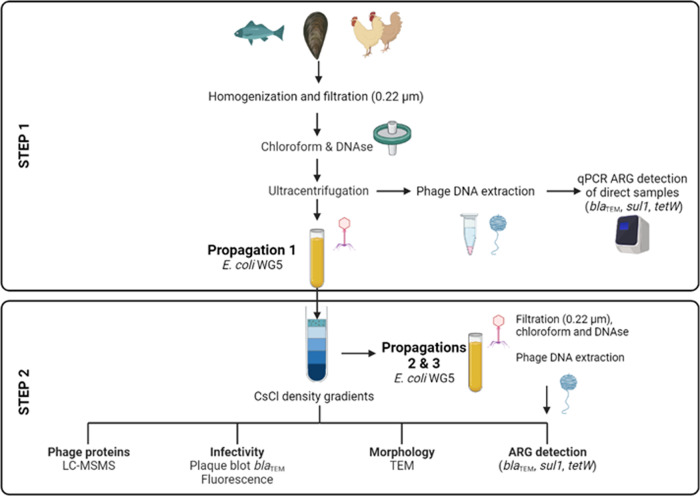
Schematic representation of the protocol performed in this study. Step 1 before and Step 2 after purification by CsCl density gradient centrifugation. Figure created in Biorender.com.

The easily visible gray bands corresponding to phages [18, 19] were collected in 0.5 ml volume by puncturing the tube. Bands were then dialyzed using prepared dialysis membranes (Molecular weight cutofffrom 12–14 kDa) (Medicell Membranes Ltd, London, UK) in dialysis buffer (Tris 0.1 M, EDTA 0.2 mM, pH 8) for 1 h. The dialysis buffer was replaced with fresh buffer and further dialyzed for 18 h with magnetic stirring. The CsCl purified phage suspensions were used for the subsequent experiments: ARG quantification, two additional propagation rounds, formation of plaque of lysis, electron microscopy observation, particle infectivity, and proteomic analysis (Fig. 1).

### ARG detection

Phage suspensions, obtained directly from samples or after propagation in *E. coli* WG5 were processed to extract the DNA within the phage particles. After treating the samples with DNAse, an aliquot was taken and used in an ARG amplification control assay, in which negative results confirmed the removal of non-packaged DNA. Then, to break the capsids, suspensions were digested with proteinase K (20 mg/ml) in 250 μl of proteinase K buffer for 1 h at 55 °C [18]. The encapsidated DNA was extracted by phenol-chloroform (1:1) (v/v) and chloroform treatment. Phenol and chloroform were removed using 2 ml Phase Lock Gel tubes (Eppendorf. Hamburg, Germany) and centrifugation at 16,000 × *g* for 5 min. Extracted DNA was precipitated using 100% ethanol and 3 M sodium acetate, resuspended in 50 µl of ultrapure water, and quantified with a Qubit Fluorometer (Life Technologies. Carlsbard, CA, US).

The ARGs in the phage DNA fractions were quantified with a quantitative real-time PCR (qPCR) assay using TaqMan hydrolysis probes, performed with a StepOne Real Time PCR System in a 20 μl reaction mixture with the TaqMan Environmental Master Mix 2.0 (Applied Biosystems, Foster City, CA, US). The reaction contained 9 μl of the sample DNA or standards with known DNA concentration. The results were analyzed with the Applied Biosystems StepOne Instrument program. Three ARGs frequently found in environmental bacterial populations [20, 21] and previously shown to be abundant in the types of food sample analyzed [10, 15] were targeted: *bla*_TEM_, which confers resistance to β-lactam antibiotics; *sul1*, to sulfonamides, and *tetW* to tetracycline. The primers used in the study are shown in Table S1.

For quantification, serial dilutions of gBlocks Gene Fragments (Integrated DNA Technologies, Coralville, IA, US) for each ARG were used to generate the standard curves in each qPCR assay. All samples were run in triplicate (including the standards and negative controls). The number of gene copies was defined as the mean of the triplicate data obtained. To evaluate ARG abundance, the gene copies were calculated with the standard curves. The standards were also used as positive controls.

To ensure that only ARGs from the phage particles were detected, several controls were performed, including ARG amplification of cultures of the host strain *E. coli* WG5 without the presence of phages and controls to confirm the DNase activity and the digestion of plasmid DNA [22]. Briefly, after filtration and treatment with chloroform and DNase, and before proteinase K digestion, an aliquot of each sample was tested to confirm the absence of each ARG by qPCR as described above and the bacterial 16 S rRNA gene (Table S1) by qPCR amplification using the Power SYBR Green PCR Master Mix (Thermo Fisher Scientific. Waltham, MA, US). Only samples negative for the respective ARGs and the 16 S rRNA gene were considered free of non-encapsidated DNA and included in the analysis.

After a first round of propagation, the cultures were propagated again in two additional rounds under the same conditions described above and ARGs were enumerated again by qPCR after each propagation step.

### Plaque blot analysis

To determine the presence of plaques of lysis generated by infectious phages containing ARG, 10 ml of the supernatant of the propagation 1 were purified by filtration and chloroform-treated as described above. Ten-fold dilutions of the filtrates were analyzed in duplicate for the presence of phages following the ISO standard method [23] that uses *E. coli* strain WG5 as the bacterial host. Plates were incubated at 37 °C for 18 h.

The DNA fragment of the *bla*_TEM_ resulting from amplification with the corresponding PCR primers (Table S1) was labeled by incorporating digoxigenin-11-deoxy-uridine-triphosphate (Roche Diagnostics. Barcelona, Spain) during PCR as described [24] Plaque hybridization was performed using nylon N + membranes (Hybond N+, Amersham Pharmacia Biotech, Spain) as previously described [18]. Stringent hybridization was achieved with the DIG DNA Detection Kit (Roche Diagnostics, Barcelona, Spain) according to the manufacturer’s instructions.

### SYBR Gold staining of phage suspensions

The concentrated CsCl phage suspensions were stained with SYBR Gold (Molecular probes, Thermo Fisher Scientific) as previously reported [25]. Briefly, 20 µl of SYBR Gold 100X was added per ml of phage particle suspension. Suspensions were gently mixed and incubated for 1 h in the dark. Afterward, they were washed four times with 0.01 M MgSO_4_ using Amicon Ultra-15 Centrifugal Filters 50 K (Merck Millipore) to remove the excess of SYBR Gold and suspended in a final volume of 1 ml using 0.01 M MgSO_4_.

### Fluorescence microscopy observations

A 1/10 dilution of a mid-log exponential culture of *E. coli* WG5 grown in LB broth to an OD_600_ of 0.2 (10^7^ CFU/ml) was mixed with 500 μl of DAPI (20 μg/ml) (Sigma Aldrich) and incubated for 15 min at room temperature in the dark. The mixture was gently centrifuged (4000 × *g* for 2 min) twice to remove the extra staining and the pellet was suspended in 0.01 M MgSO_4_.

One hundred μl of DAPI stained bacterial cells were then centrifuged and resuspended in 100 μl of SYBR Gold-stained phage suspension. The mixture was incubated at 37 °C for 1 h and then fixed with 50 μl of 4% paraformaldehyde solution for 5 min. To remove excess paraformaldehyde, the suspensions were washed twice and suspended in a final volume of 50 μl. The mixture was observed under a confocal fluorescence microscope Zeiss LSM 880 (Zeiss, Jena, Germany).

### Electron microscopy observations

Seven µl of the CsCl-concentrated phage suspensions were dropped onto copper grids with carbon-coated Formvar films and negatively stained with 2% ammonium molybdate (pH 6.8) for 2 min. Phages were visualized using a Jeol 1010 Transmission Electron Microscope (TEM) (JEOL Inc. Peabody, MA US) operating at 80 kV.

### Proteomic analysis

One hundred µl of the CsCl-concentrated phage suspensions was used for proteomic analysis.

### Sample preparation and protein digestion

#### SDS-PAGE clean-up-

Total protein quantification was carried out with a Pierce BCA Protein assay (Thermo Fisher), and proteins were cleaned of interfering substances for mass spectrometry by running the sample in a SDS-PAGE system (BioRad. Hercules, CA, US). Then, proteins were fixed for 1 h in a solution of 10% acetic acid/40% ethanol/50% Milli-Q water (v/v/v) and washed three times with Milli-Q water.

#### In-gel digestion-

The acrylamide gels were sliced into cubes and the slices were washed with 50 mM ammonium bicarbonate (NH_4_HCO_3_) and acetonitrile (ACN), reduced with 20 mM DTT in 50 mM NH_4_HCO_3_ for 60 min at 60 °C, and alkylated with 55 mM iodoacetamide in 50 mM NH_4_HCO_3_ for 30 min at 25 °C in the dark. Afterward, the samples were digested with trypsin (Sequence Grade Modified Trypsin (Promega, Wisconsin, US)) for 2 h at 37 °C. Then, a new aliquot of trypsin was added and the digestion was allowed to continue overnight at 37 °C. The resulting peptide mixtures were extracted from the gel matrix with 5% formic acid (FA) in 50% ACN and 100% ACN, dried and stored at −20 °C until the LC-MS analyses.

### LC-MSMS analysis

The dried peptide mixtures were analyzed in a nanoAcquity liquid chromatographer (Waters Corp. Milford, MA, US) coupled to an LTQ-Orbitrap Velos (Thermo Fisher Scientific) mass spectrometer (MS) after resuspension in 1% FA solution. The 15 most abundant peptides (minimum intensity of 500 counts) were selected from each MS scan and then fragmented in the linear ion trap using collision induced dissociation with helium as the collision gas (38% normalized collision energy). Generated raw data files were collected with Thermo Xcalibur (v.2.2) (Thermo Fisher Scientific).

### Data analysis

A database was created by merging all entries in the public database SwissProt (v.9/4/21) with all entries for viruses in the public database TrEMBL (taxonomy 10662 v.27/4/21).

Mass spectrometry data were analyzed with the Sequest HT search engine using Thermo Proteome Discoverer (v.1.4.1.14) (Thermo Fisher Scientific) against the customized database. To improve the sensitivity of the database search, Percolator (semi-supervised learning machine) was used to discriminate between correct and incorrect peptide spectrum matches. Percolator assigns a *q* value to each spectrum, which is defined as the minimal false discovery rate (FDR) at which the identification is deemed correct. Results were filtered so only proteins identified with at least 2 peptides (FDR ≤ 5%) were included.

## Results and discussion

### Microbiological parameters of the samples

The morphological types of the colonies grown in TSA were quite homogeneous and the majority of the aerobic bacteria were found to be resistant to amp (Table S2) with differences close to 1 log_10_ units between the number of colonies grown in the absence or presence of ampicillin. Dark blue colonies on Chromocult agar were identified as *E. coli*, whereas *E. coli* and amp-resistant *E. coli* (dark blue colonies grown on Chromocult agar containing amp) were detected only in chicken (Table S2). The differences between the number of colonies grown with or without amp in chicken, showed also that a large fraction of the *E. coli* detected was amp resistant. Microbial indicators in these samples were in accordance with previous analyses in similar type of samples [10, 15]. The presence of resistant bacteria points towards the bacterial populations as possible sources of ARGs in the samples.

### ARG detection in the phage suspensions before and after propagation

**Fig. 2:**
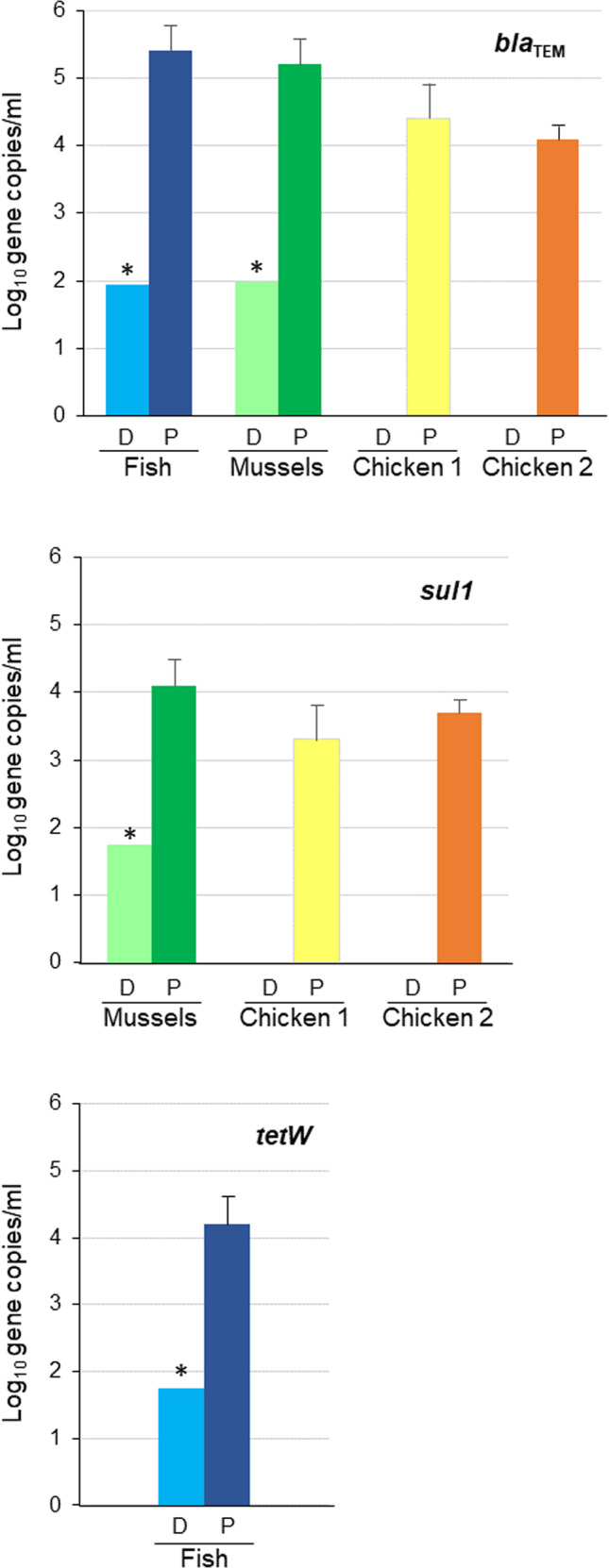
ARG abundance in the phage fraction of the samples. Abundance (log_10_ gene copies/ml) of *bla*_TEM_, *sul1*, or *tetW* in the DNA fraction of the purified particles obtained in Step 2 from samples of fish, mussels, and chicken 1 and 2 in direct samples (D) and after the first propagation (P). *tetW* was only detected in fish samples and *sul1* in mussels, and chicken 1 and 2. Asterisk defines those ARGs detected, but with values below the limit of quantification, therefore their abundance could only be estimated. Each column represents the average value of three independent experiments and error bars represent the standard deviation.

Chicken samples had undetectable levels of the three ARGs (*bla*_TEM_, *sul1*, *tetW*) when analyzed before the enrichment culture (Direct samples (D) in Fig. 2). Mussels and fish direct samples showed amplification of the different ARGs (Fig. 2). Even if the results of ARGs detected fell below the limit of quantification (LOQ) of the standard curves used (Table S1) and therefore they could not be enumerated properly, they were present in the original mussels and fish samples. After a first round of propagation on *E. coli* WG5 and purification by CsCl density gradient centrifugation, a single band was identified and recovered (Fig. 1). The band was located at a density of 1.3–1.5 (Step 2 in Fig. 1), which corresponds to the buoyant density of most bacteriophages, particularly those belonging to the *Caudovirales* order [19, 26]. The band was recovered, dialyzed, and treated with DNase to remove any non-encapsidated DNA. After inactivation of the DNAse, the qPCR amplification of the three target ARGs and of 16 S rRNA, performed to control the removal of non-packaged DNA, was negative. In the subsequent steps of the protocol, the capsids were broken, the packaged DNA purified, and the ARGs again amplified by qPCR.

After the first round of propagation, *bla*_TEM_ and *sul1* were detected in mussels and chicken, and *bla*_TEM_ and *tetW* in fish (Propagated samples (P) in Fig. 2). In previous studies, these were the ARGs found in the highest densities in the same type of food samples [10, 15], hence their selection for the current work. The absence of ARGs in the host strain WG5 used for propagation was confirmed in the control culture without phages, in accordance with previous studies showing that *E. coli* WG5 does not carry any ARGs in its genome [17].

The non-detection of ARGs in the directly analyzed chicken samples was probably because the densities of ARG-carrying phage particles were below the detection limit. In contrast, the three ARGs were detected after the first propagation step, indicating that some of these particles were able to propagate after infecting the *E. coli* strain WG5. An alternative explanation is that after the CsCl density gradient centrifugation the particles were better purified, with a reduced presence of possible PCR inhibitors, resulting in a higher detection limit. However, this explanation is unlikely, as in previous studies ARGs were detected at high densities in the same type of samples using a very similar protocol without CsCl purification [10, 15].

The levels of propagation observed (increase of 3–5 log_10_ units of gene copies/ml after propagation 1) suggest that not all the ARG-containing phages in the direct samples were able to propagate on *E. coli* WG5, otherwise higher titers could be expected after 18 h of incubation. It is also possible that some transducing particles (i.e., unable to propagate) were present in the samples, but were unobserved as their numbers were below the detection limit.

These observations indicate that at least part of the phage particles in the original samples were infectious and they were the source of ARGs detected after the propagation step. Propagation of ARG-containing phages has been described in previous studies with meat and fish samples [10, 27], vegetables [28], and dairy products [29]. In the process of attachment and injection, genes are incorporated in the recipient cells, where they may undergo legitimate or illegitimate recombination or not. Although transducing particles can inject DNA, only complete phages can replicate.

Phages in the supernatant of the propagation round 1 of the samples generated plaques of lysis onto strain *E. coli* WG5 at densities ranging from 10^6^ in fish to 10^8^ in mussels and chicken. However, plaque blot hybridization with the *bla*_TEM_-DIG-labeled probe did not reveal positive plaques for this ARG in any sample. Considering that *bla*_TEM_ was the most abundant ARG detected, these results suggested that phages carrying *bla*_TEM_ in the samples were unable to generate plaques of lysis. It cannot be excluded that infectious phages carrying *bla*_TEM_ were present but could be in a too low a proportion compared to other virulent phages in the suspension, preventing their detection in the dilutions performed to obtain separate plaques.

The CsCl purified phage particles obtained after propagation round 1 were used to further propagate phages in two additional successive rounds (Propagation rounds 2 and 3). ARG detection was performed after these propagation steps to gain insights into the infectivity of the phage particles carrying ARGs. The number of gene copies of the three ARGs evaluated did not show any increase, but a slight decrease corresponding to 1/10 dilution factor in propagation 2 and 1/100 dilution factor in propagation 3 (data not shown), compared with the values of propagation 1 (Fig. 2). This lack of increase in the ARG copy numbers suggested that the ARG-containing particles generated after the first propagation round were unable to propagate in the host strain, at least the great majority of them. This might happen because other lytic phages have been selected, causing the lysis of the host strain before ARG-carrying particles could propagate. Another explanation is that the ARG-carrying particles generated in the first propagation round were transducing particles containing only bacterial DNA, hence unable to propagate as discussed below.

### Attachment of phage particles to the host cells and DNA injection

**Fig. 3:**
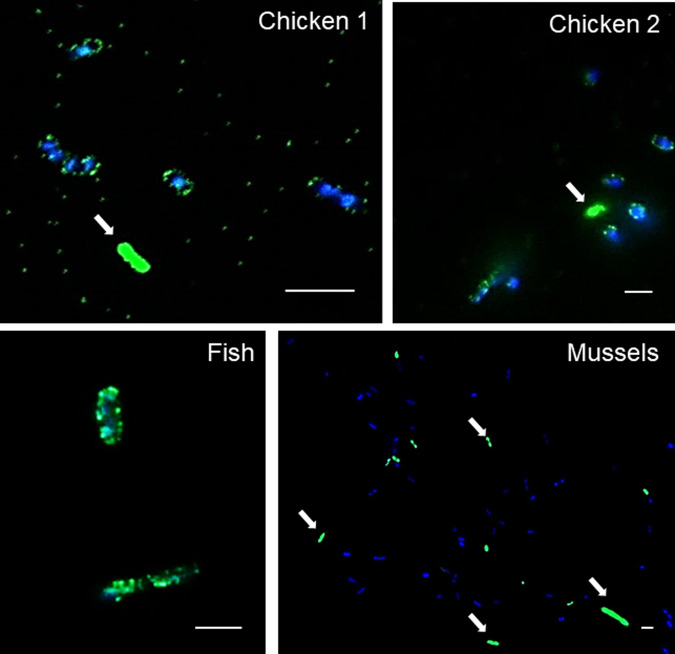
Infectivity of the phage particles. Confocal fluorescence microscopy images of DAPI-stained *E. coli* strain WG5 and SYBR Gold-stained purified particles obtained in Step 2 from fish, mussels, chicken 1 and 2 samples. The mixture was incubated at 37 °C for 1 h. Arrows show presumably phage-infected bacterial cells with a complete fluorescent signal. Scale bar: 5 µm.

The first requirement for ARG transduction is the ability of phage particles to attach themselves to a suitable recipient cell and inject their DNA, regardless of whether they are complete phages that can propagate or phage capsids containing only bacterial DNA. According to the results obtained thus far, at least some of the ARG-carrying particles had the capacity to propagate, as their numbers increased and became detectable after the first propagation round. To visualize their attachment to cells, the phage particles obtained after CsCl density gradient centrifugation (Step 2, Fig. 1) were labeled with SYBR Gold and used to infect DAPI-stained *E. coli* WG5 cells. The results showed phage particles from all the analyzed samples anchored to the surface of cells (Fig. 3), which is the first stage of phage infection. In some cases, bacterial cells were stained and visualized in green (Fig. 3, white arrows), attributable to a possible injection of the phage DNA [30]. Other cells remained blue, indicating that phages did not become attached or inject their DNA (for example, in mussels; see Fig. 3).

Transduction of ARGs was not achieved in this study despite multiple attempts and the evidence that some of the ARG-carrying phage particles were infectious. ARG transduction may be hindered by different factors: *E. coli* WG5 may not be the most suitable recipient host; the lack of recombination events that would allow maintenance of the transferred DNA inside the recipient host; or the presence of other lytic phages that may infect and lyse the recipient host before successful transduction can occur.

### Presence of phages in the samples

**Fig. 4:**
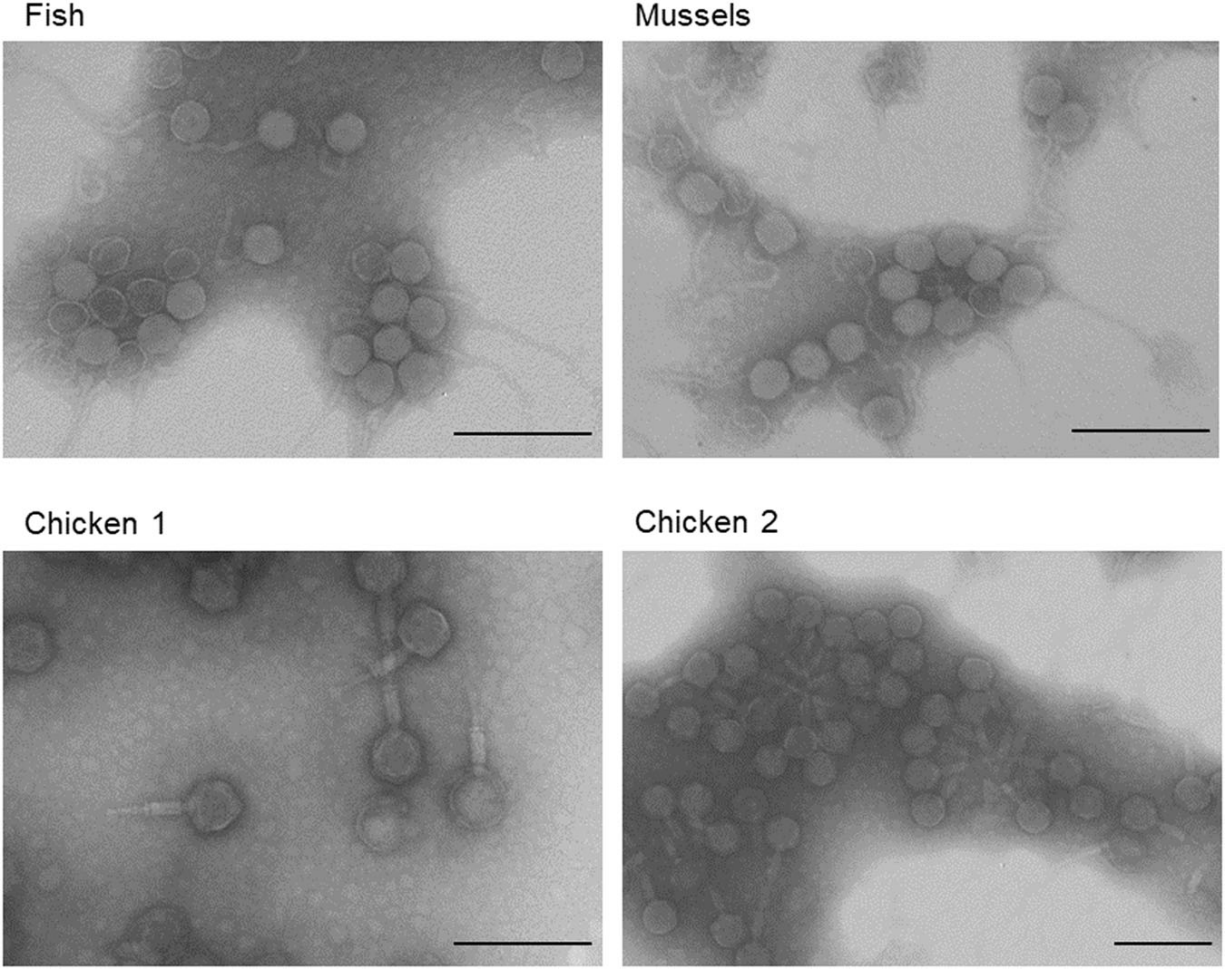
Morphology of the phage particles. Transmission electron microscope images of the most abundant purified particles obtained in Step 2 from samples of fish, mussels, and chicken 1 and 2 samples. Scale bar: 200 nm.

Phage presence in the suspensions was verified by TEM (Fig. 1, Step 2), which showed siphophages in fish and mussel samples and myophages in both chicken samples (Fig. 4). The propagation step seemed to have selected one predominant morphological type in each sample, although Caudovirales phages of other morphologies were also observed (a few myophages in mussels and a few siphophages in chicken). Also, tailless icosahedral capsids of 50–55 nm, which may have been podophages or siphophages whose tails were lost during the process, were visualized in all samples (data not shown). No structures compatible with OMVs were observed.

One of the criticisms aimed at studies reporting ARG-containing phage particles concerns the potential interference of OMVs with the results. OMVs contain components of the bacterial cytoplasm, as well as DNA, including ARGs [31, 32]. Small ARG-containing OMVs can pass through 0.22 µm membrane filters and, if no additional steps are taken for their removal, the protocol used for phage DNA extraction may also extract OMV-associated DNA, leading to the false detection of ARG-containing phages.

**Fig. 5:**
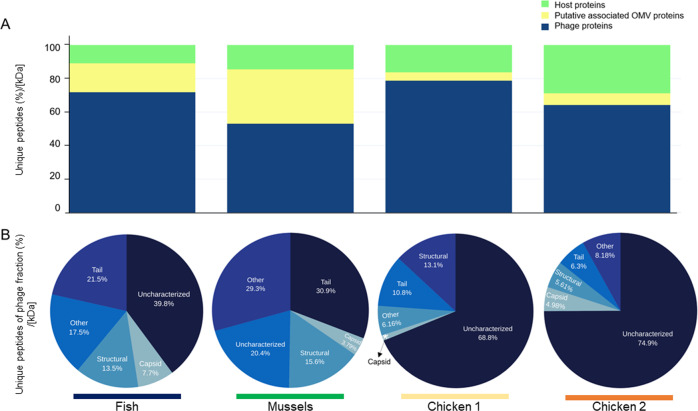
Proteomic analysis of the phage particles. Proteomic analysis of purified particles obtained in Step 2 from samples of fish, mussels, and chicken 1 and 2. **A** Percentage of unique proteins identified as phage, membrane or other proteins in the bacterial host. **B** Percentage of unique phage proteins identified as structural, tail, capsid, or other phage proteins or uncharacterized. In *Y*-axis, the percentage of unique peptides has been normalized using the molecular mass of each peptide in KDa and is indicated as [KDa]. Normalization allows the comparison between proteins of different size.

Chloroform is commonly used to prepare phage lysates, as it disrupts the lipid membrane of bacteria [33]. Organic solvents, such as chloroform [23] or butanol [34], are also used for phage purification as they can break apart OMVs and release the DNA content, which is subsequently digested by the DNAse. In addition to direct observations by TEM, for further confirmation that the OMVs had been efficiently removed, the protein fractions of the purified particles (obtained in Step 2 of the protocol) were analyzed by mass spectrometry. Peptides were identified using a custom database that included all the relevant entries from SwissProt and TrEMBL. The most abundant proteins in all samples belonged to phages; the percentages of unique peptides associated with phages were 71.8% (fish), 52.9% (mussels), 78.7% (chicken 1), and 64.1% (chicken 2) (Fig. 5). In contrast, membrane proteins accounted for only 16.2% (fish), 21.4% (mussels), 4.2% (chicken 1), and 3.8% (chicken 2) of the proteins in the purified particles, indicating a low presence of OMVs. The results were normalized by protein size (KDa) to avoid the overrepresentation of large versus small proteins. The rest of the identified proteins belonged to bacterial hosts and were not of cell membranes (Table S3). Among the viral proteins, the highest proportion identified in all the samples were uncharacterized phage proteins, except in the viral suspension of mussels, where tail proteins predominated (Fig. 5). The second most abundant overall were tail or structural proteins.

The abundance of “unknown” sequences has been widely reported in genomic analyses of viral communities in nature. However, new approaches based on metaproteomics and metagenomics have allowed tentative functions to be assigned to viral genomic sequences and previously unknown virion-associated proteins in environmental samples [35], leading to 67% of the studied metaproteomes being tentatively identified as capsid proteins. Accordingly, it is likely that most of the uncharacterized phage proteins found in our samples are also capsid proteins. Their homology indicates they belonged to Caudovirales phages infecting *Enterobacteriaceae*, which is in accordance with phages able to infect *E. coli* WG5. The obtained matches could be biased by the entries in the databases, which are enriched with phages of enterobacteria, or the presence of polyvalent phages able to infect different members of the *Enterobacteriaceae* family, as well as by the high protein conservation between phages infecting different genera of enterobacteria [36, 37]. These results, together with the low content of membrane proteins in the samples, support the predominance of phage particles in the phage suspensions, confirming that the protocol efficiently removed the OMVs. The membrane proteins detected might derive from membrane fragments rather than complete OMVs. Overall, it seems unlikely that OMVs contributed significantly to the ARG content in the purified suspensions.

ARG mobilization by phages has only recently been considered as a significant mechanism responsible for the emergence of new multi-resistant strains. Phage capsids form a shield that can protect DNA content and prolong DNA persistence in extracellular environments [11]. Although ARG dissemination in phage particles does not necessarily result in their transduction, it is the first step towards this outcome. Transducing particles can be difficult to isolate and ARG transduction may be hindered by different factors as discussed above. But if the generation of DNA-containing phage particles is as successful as suggested by recent studies [38], the strategy seems to enhance the probability of transduction by increasing the number of particles potentially able to transduce. Another question is whether the particles mobilizing ARGs are the product of lateral or generalized transduction events, which generate phage capsids containing only bacterial DNA, or if they are complete infectious phages harboring one ARG, the product of specialized transduction. Our results suggest that the phage contribution to ARG mobilization probably involves both types of particles. Gene transfer agents [39] are not considered here since they have not been described in *E. coli* genera and because strain WG5 in this study does not possess phage genes in its genome [17].

The fact that only the first, but not subsequent propagation rounds, resulted in an increase in the ARG copy numbers, together with the lack of lytic plaques positive for the ARGs discards the presence of temperate phages containing ARG and points towards transducing particles containing only bacterial DNA as the more likely contributors to the numbers of ARGs detected in the phage DNA fraction here and in previous studies [12, 14, 40–42]. Another explanation would be the presence of elements such as the phage-like plasmids [43–45], that can be present in the samples as virions, being able to enter in the host strain, replicate as plasmids and generate new virions carrying the ARG. After release from the host, by lysis or by the chloroform treatment, they could be detected in the first propagation step being the responsible for the increase in the ARG copy numbers observed. However, if the virions generated in this first round are transducing particles or defective virions, these might be unable to generate plaques of lysis or to propagate successive infective rounds 2 and 3. Another possibility could be the co-infection by two phages in the first propagation round [46, 47], one helper phage that could package the DNA of a second defective phage or transducing particle carrying the ARG, both present in the original sample. This co-infection would also have accounted for the generation of ARG-carrying phage particles in the first step of propagation. However, these particles would be unable to generate plaques or lysis and, in addition, the differential propagation of phages in the first round of propagation would have eliminated the presence of the helper phage in the suspension, reducing the possibility of co-infection and making ARG-transducing particles unable to propagate in its absence in propagation rounds 2 and 3. Notwithstanding, what seems clear is that the results were not affected by contamination by non-packaged DNA, as the ARGs were not detected before the capsids were broken, nor were the target ARGs mobilized by OMVs to any great extent. The removal of OMV-associated DNA was clearly effective, as the enrichment procedure of the protocol would have generated many OMVs from the *E. coli* strain, in addition to those naturally present in the samples. In comparison, the contribution of phages or phage capsids to ARG mobilization in the viromes is clearly demonstrated, in confirmation of previous studies [9, 40–42, 48–51].

In the search to find solutions that could reduce or minimize the emergence of new resistant strains, acquiring further knowledge about the elements contributing to ARG dissemination and the underlying mechanisms is essential. To do so, it is necessary to confirm the reliability of the protocols used to obtain data about ARG-carrying phage particles in the viromes, which was the goal of this study.

## Supplementary information

Supplementary Tables S1 and S2

Supplementary Table S3

## Data Availability

All data are available in the main text or the supplementary online materials.
