# Supplementary material for: Dominance of phage particles carrying antibiotic resistance genes in the viromes of retail food sources
Source: ISME J. 2022 Oct 26;17(2):195–203. doi: 10.1038/s41396-022-01338-0 (PMC9860054; doi:10.1038/s41396-022-01338-0)
Supplement: Supplementary file 1 — Supplementary Tables S1 and S2 [file 41396_2022_1338_MOESM1_ESM.docx]

**Table S1.-** Oligonucleotides used in this study

| **Target gene** | **Oligonucleotide** | **Sequence** | **Amplimer (bp)** | **LOQ (Ct)** | **Ref.** |
| --- | --- | --- | --- | --- | --- |
| *bla*_TEM_ qPCR | UP | CACTATTCTCAGAATGACTTGGT | 85 | 35.2 | [1] |
|  | LP | TGCATAATTCTCTTACTGTCATG |  |  |  |
|  | TaqMan TEM | 6FAM-CCAGTCACAGAAAAGCATCTTACGG-MGBNFQ |  |  |  |
| *bla*_TEM_ PCR | UP | CTCACCCAGAAACGCTGGTG | 569 | - | [2] |
|  | LP | ATCCGCCTCCATCCAGTCTA |  |  |  |
| *sul1* qPCR | UP | CCGTTGGCCTTCCTGTAAAG | 67 | 34.8 | [3] |
|  | LP | TTGCCGATCGCGTGAAGT |  |  |  |
|  | TaqMan sul1 | 6FAM-CGAGCCTTGCGGCGG-MGBNFQ |  |  |  |
| *tetW* qPCR | UP | GACGGACACCATGTTTTTGGA | 62 | 34.8 | [4] |
|  | LP | AGGAAGTGACTGCCGCTTGA |  |  |  |
|  | TaqMan tetW | 6FAM-AGCGTGGGATTACCA- MGBNFQ |  |  |  |
| 16S rRNA qPCR | 338F | ACTCCTACGGGAGGCAGCAG | 236 |  | [5] |
|  | 518R | ATTACCGCGGCTGCTGG |  |  |  |

LOQ: Limit of quantification

**Tabla S2.-** Bacterial indicators (CFU/25 g) in the samples of the study

| **Microorganism** |  | **Fish** | **Mussel** | **Chicken 1** | **Chicken 2** |
| --- | --- | --- | --- | --- | --- |
| Total aerobic bacteria | Media | 2.8 10^5^ | 6.0 10^7^ | 2.0 10^6^ | 6.0·10^5^ |
|  | *SD* | *1.1 10^5^* | *4.0 10^6^* | *4.3·10^3^* | *1.2·10^3^* |
| Total aerobic bacteria amp^R^ | Media | 6.3 10^4^ | 2.4 10^6^ | 8.8 10^5^ | 2.0·10^4^ |
|  | *SD* | *1.4 10^4^* | *1.8 10^6^* | *4.2 10^4^* | *6.0·10^3^* |
| *E. coli* | Media | 0 | 0 | 1.3 10^3^ | 1.0 10^3^ |
|  | *SD* | *-* | *-* | *3.3 10^1^* | *8.0 10^1^* |
| *E. coli* amp^R^ | Media | 0 | 0 | 1.0 10^2^ | 9.0 10^1^ |
|  | *SD* | *-* | *-* | *4.4 10^1^* | *2.0 10^0^* |

**Supplementary references**

1. Lachmayr KL, Kerkhof LJ, Dirienzo AG, Cavanaugh CM, Ford TE. Quantifying nonspecific TEM beta-lactamase (blaTEM) genes in a wastewater stream. *Appl Environ Microbiol* 2009; **75**: 203–11.

2. Colomer-Lluch M, Jofre J, Muniesa M. Antibiotic resistance genes in the bacteriophage DNA fraction of environmental samples. *PLoS One* 2011; **6**: e17549.

3. Calero-Cáceres W, Melgarejo A, Colomer-Lluch M, Stoll C, Lucena F, Jofre J, et al. Sludge as a potential important source of antibiotic resistance genes in both the bacterial and bacteriophage fractions. *Environ Sci Technol* 2014; **48**: 7602–11.

4. Blanco-Picazo P, Roscales G, Toribio-Avedillo D, Gómez-Gómez C, Avila C, Ballesté E, et al. Antibiotic resistance genes in phage particles from Antarctic and Mediterranean seawater ecosystems. *Microorganisms* 2020; **8**: 1293.

5. Weisburg WG, Barns SM, Pelletier DA, Lane DJ. 16S ribosomal DNA amplification for phylogenetic study. *J Bacteriol* 1991; **173**: 697–703.
