# Supplementary material for: Dominance of phage particles carrying antibiotic resistance genes in the viromes of retail food sources
Source: ISME J. 2022 Oct 26;17(2):195–203. doi: 10.1038/s41396-022-01338-0 (PMC9860054; doi:10.1038/s41396-022-01338-0)
Supplement: Supplementary file 2 — Supplementary Table S3 [file 41396_2022_1338_MOESM2_ESM.pdf]

Accession	Description	Score	Coverage	# Proteins	# Unique Pe	# Peptides	% of Unique	% of Peptide	% of Unique	% of Peptide	# PSMs	# AAs	MW [kDa]	calc. pI	% of Unique Peptides/[kDa] (Total	% of Peptides/[kDa] (Total)
A0A2I6PGP2	Major capsid protein OS=Salmonella phage BPS15S6 OX=2060121 GN=BPS15S6_11 PE=3 SV=1 - [A0A2I6PGP2_9CAUD]	570,155441	58,15	1	4	21	0,85470085	2,27027027	0,02059132	0,054695	199	368	41,5078184	5,74462891	**1,864508166**	1,75051225
A0A2S1GPP5	Putative structural protein OS=Enterobacteria phage vB_EcoM_IME338 OX=2163888 PE=4 SV=1 - [A0A2S1GPP5_9CAUD]	461,879526	55,71	9	17	32	3,63247863	3,45945946	0,06294029	0,05994237	141	508	57,7130863	6,37939453		
A0A6B9WZI9	Tail tapemeasure protein OS=Escherichia phage pinkbiff OX=2696440 GN=pinkbiff_21 PE=4 SV=1 - [A0A6B9WZI9_9CAUD]	390,625679	45,24	1	4	31	0,85470085	3,35135135	0,01065481	0,04177839	105	736	80,2173498	9,67138672		
A0A455XBP6	phage tail tape measure OS=Escherichia phage L27 OX=2562890 PE=4 SV=1 - [A0A455XBP6_9CAUD]	380,828899	54,42	1	3	39	0,64102564	4,21621622	0,0079953	0,05258747	109	735	80,175295	9,65673828		
A0A0H3UCS	Major capsid protein OS=Escherichia phage HY02 OX=1527531 GN=HY02_058 PE=3 SV=1 - [A0A0H3UCS0_9CAUD]	375,196736	58,15	1	1	19	0,21367521	2,05405405	0,00513717	0,04938346	113	368	41,5939644	5,77001953		
A0A6B9XCT5	Major capsid protein OS=Escherichia phage warpig OX=2696461 GN=warpig_95 PE=3 SV=1 - [A0A6B9XCT5_9CAUD]	375,196736	63,86	1	1	19	0,21367521	2,05405405	0,00515279	0,04953365	113	368	41,4678487	5,63037109		
A0A481W3F	Tail length tape-measure protein OS=Escherichia phage PHB11 OX=2530036 PE=4 SV=1 - [A0A481W3F9_9CAUD]	362,587187	55,1	1	3	39	0,64102564	4,21621622	0,00798564	0,05252394	105	735	80,2722719	9,67138672		
A0A455XHL7	phage protein OS=Escherichia phage L27 OX=2562890 PE=4 SV=1 - [A0A455XHL7_9CAUD]	322,148818	66,87	2	2	23	0,42735043	2,48648649	0,00805727	0,04688024	97	489	53,0391207	4,66552734		
A0A6B9WQ1	Baseplate assembly protein OS=Escherichia phage skuden OX=2696443 GN=skuden_43 PE=4 SV=1 - [A0A6B9WQ14_9CAUD]	288,274184	66,46	2	3	23	0,64102564	2,48648649	0,01207271	0,04682905	86	489	53,0970898	4,61474609		
A0A384SJI6	Tail fibers protein OS=Enterobacteria phage XTG1 OX=1651201 PE=4 SV=1 - [A0A384SJI6_9CAUD]	286,823802	29,97	1	1	18	0,21367521	1,94594595	0,00248917	0,02266898	91	794	85,8417953	8,77783203		
A0A6B9XFQ2	Putative tail protein OS=Escherichia phage radambza OX=2696441 GN=radambza_56 PE=4 SV=1 - [A0A6B9XFQ2_9CAUD]	282,961296	44,71	1	12	12	2,56410256	1,2972973	0,08181677	0,04139487	76	293	31,3395703	5,02099609		
A0A384SJ98	Tail fibers protein OS=Enterobacteria phage KhF2 OX=1651204 PE=4 SV=1 - [A0A384SJ98_9CAUD]	266,785674	26,39	1	2	18	0,42735043	1,94594595	0,00498625	0,02270497	86	792	85,7057226	8,49951172		
A0A6B9WPE	Tail fibers protein OS=Escherichia phage skuden OX=2696443 GN=skuden_39 PE=4 SV=1 - [A0A6B9WPE2_9CAUD]	257,581438	24,33	1	3	18	0,64102564	1,94594595	0,00765295	0,02323186	88	781	83,7619343	8,58740234		
A0A2S1GPS1	Tail fiber protein OS=Enterobacteria phage vB_EcoM_IME338 OX=2163888 PE=4 SV=1 - [A0A2S1GPS1_9CAUD]	240,493549	22,86	1	4	18	0,85470085	1,94594595	0,01003028	0,02283652	78	783	85,2120239	6,87353516		
A0A3G8F4M	Major capsid protein (Fragment) OS=Escherichia phage VNV OX=2488710 PE=4 SV=1 - [A0A3G8F4M3_9VIRU]	214,377004	54,17	1	2	8	0,42735043	0,86486486	0,02664768	0,05392915	50	144	16,0370566	7,59130859		
A0A6B9M30	Tail fiber protein OS=Shigella phage Z31 OX=2675202 PE=4 SV=1 - [A0A6B9M307_9CAUD]	198,247812	25,79	1	2	18	0,42735043	1,94594595	0,00497566	0,02265675	70	795	85,8881497	8,86572266		
A0A482MR9	Tail fiber OS=Escherichia phage vB_EcoM_LMP33 OX=2491664 PE=4 SV=1 - [A0A482MR91_9CAUD]	179,12171	20,78	2	2	16	0,42735043	1,72972973	0,00493533	0,01997607	63	794	86,5900727	8,04541016		
A0A482JGU1	Uncharacterized protein OS=Shigella phage Silverhawkium OX=2530185 GN=Silverhawkium_gp81 PE=4 SV=1 - [A0A482JGU1_9CAUD]	164,812734	29,1	1	1	16	0,21367521	1,72972973	0,00385681	0,03122136	52	488	55,4021215	6,31591797		
A0A384T024	Tail fiber protein OS=Enterobacteria phage KhF3 OX=1651200 PE=4 SV=1 - [A0A384T024_9CAUD]	150,392019	41,27	3	10	10	2,13675214	1,08108108	0,05100214	0,02580432	54	395	41,8953453	6,96142578		
A0A482MRM	Uncharacterized protein OS=Escherichia phage vB_EcoM_LMP25 OX=2491663 PE=4 SV=1 - [A0A482MRM9_9CAUD]	125,443554	59,04	1	8	8	1,70940171	0,86486486	0,09384935	0,0474827	37	166	18,2143167	8,14794922		
A0A4Y5NX56	Uncharacterized protein OS=Salmonella phage DaR-2019a OX=2571168 GN=FFZ30_0023 PE=4 SV=1 - [A0A4Y5NX56_9CAUD]	117,398192	49,82	8	13	13	2,77777778	1,40540541	0,08778159	0,04441274	36	285	31,6441945	5,79541016		
A0A6B9SPQ	Uncharacterized protein OS=Escherichia phage JN01 OX=2692737 PE=4 SV=1 - [A0A6B9SPQ7_9CAUD]	110,11253	72	1	6	9	1,28205128	0,97297297	0,0940883	0,07140539	39	125	13,6260432	5,94775391		
A0A6G9LUJ8	Uncharacterized protein OS=phage NBEco005 OX=2712974 PE=4 SV=1 - [A0A6G9LUJ8_9CAUD]	101,938223	48,22	1	4	20	0,85470085	2,16216216	0,0174922	0,04425054	31	450	48,8618259	5,18603516		
A0A0E3T8X4	Uncharacterized protein OS=Escherichia phage vB_EcoM_AYO145A OX=1636202 GN=AYO145A_064 PE=4 SV=1 - [A0A0E3T8X4_9CAUD]	101,87698	42,22	1	3	19	0,64102564	2,05405405	0,01317769	0,0422256	30	450	48,6447561	5,18603516		
A0A2I6PGV5	Structural protein OS=Salmonella phage BPS15S6 OX=2060121 GN=BPS15S6_10 PE=4 SV=1 - [A0A2I6PGV5_9CAUD]	86,4972382	88,8	1	9	12	1,92307692	1,2972973	0,14053398	0,09480346	26	125	13,6840712	6,30322266		
Q6KGJ0	Peptidase_S49 domain-containing protein OS=Salmonella phage Felix O1 (isolate Felix O1-VT1) OX=1283336 PE=3 SV=1 - [Q6KGJ0_BPFO1]	78,5900934	22,73	21	12	12	2,56410256	1,2972973	0,05698082	0,02882921	25	418	44,9993978	4,67822266		
A0A0D4DA1	Uncharacterized protein OS=Salmonella phage vB_SPuM_SP116 OX=1567025 GN=HB2014_72 PE=4 SV=1 - [A0A0D4DA12_9CAUD]	74,4387329	41,54	10	12	12	2,56410256	1,2972973	0,06916041	0,03499143	23	325	37,0747177	8,89501953		
A0A291AZK3	Baseplate assembly protein OS=Shigella phage Sf18 OX=2024319 GN=Sf18_gp136 PE=4 SV=1 - [A0A291AZK3_9CAUD]	71,9664409	19,5	5	2	6	0,42735043	0,64864865	0,01230859	0,01868244	22	318	34,7196954	4,69091797		
A0A0D4DAW	Uncharacterized protein OS=Salmonella phage vB_SPuM_SP116 OX=1567025 GN=HB2014_70 PE=4 SV=1 - [A0A0D4DAW4_9CAUD]	63,7099726	33,96	4	1	8	0,21367521	0,86486486	0,00737838	0,02986449	20	265	28,9596408	8,22119141		
A0A0E3XDF0	Uncharacterized protein OS=Escherichia phage vB_EcoM_AYO145A OX=1636202 GN=AYO145A_070 PE=4 SV=1 - [A0A0E3XDF0_9CAUD]	55,2122338	43,36	7	5	5	1,06837607	0,54054054	0,08069548	0,04082755	16	113	13,239602	4,81787109		
A0A386K515	Uncharacterized protein OS=Enterobacter phage phi63_307 OX=2340711 GN=LINGLNFE_00086 PE=4 SV=1 - [A0A386K515_9CAUD]	54,1603898	7,29	6	1	3	0,21367521	0,32432432	0,00391651	0,00594463	18	480	54,557573	5,82080078		
A0A0F6R8U7	Uncharacterized protein OS=Escherichia coli O157 typing phage 15 OX=1508677 GN=ECTP15_02455 PE=4 SV=1 - [A0A0F6R8U7_9CAUD]	54,123055	38,49	4	2	9	0,42735043	0,97297297	0,01493376	0,03400053	18	265	28,6164076	7,23974609		
A0A3T0IN25	Uncharacterized protein OS=Shigella phage vB_SflM_004 OX=2500763 GN=vBSflM004_089 PE=4 SV=1 - [A0A3T0IN25_9CAUD]	51,245755	35,07	8	3	3	0,64102564	0,32432432	0,0435465	0,02203218	13	134	14,7204848	5,17333984		
A0A222Z063	Uncharacterized protein OS=Salmonella phage ST11 OX=2023990 PE=4 SV=1 - [A0A222Z063_9CAUD]	50,5696301	45,27	4	9	9	1,92307692	0,97297297	0,11388288	0,05761858	16	148	16,8864442	5,74462891		
S4TPL8	Uncharacterized protein OS=Salmonella phage FSL SP-012 OX=1173771 GN=SP012_00160 PE=4 SV=1 - [S4TPL8_9CAUD]	49,816009	67,86	1	11	11	2,35042735	1,18918919	0,10571867	0,05348793	17	196	22,232851	6,77099609		
A0A5P8D4K9	Uncharacterized protein OS=Pectobacterium phage Wc4-1 OX=2652429 PE=4 SV=1 - [A0A5P8D4K9_9CAUD]	48,4024241	10,08	2	1	5	0,21367521	0,54054054	0,00389608	0,00985603	13	486	54,8436643	6,10009766		
A0A0H3UDV	Uncharacterized protein OS=Escherichia phage HY02 OX=1527531 GN=HY02_072 PE=4 SV=1 - [A0A0H3UDV6_9CAUD]	39,8764544	35,97	6	3	3	0,64102564	0,32432432	0,04104171	0,02076489	11	139	15,6188817	5,94775391		
A0A384TF93	Uncharacterized protein OS=Enterobacteria phage XTG1 OX=1651201 PE=4 SV=1 - [A0A384TF93_9CAUD]	33,2126919	42,86	7	6	6	1,28205128	0,64864865	0,08227905	0,04162875	10	133	15,5817469	6,18896484		
A0A0E3TAX3	Uncharacterized protein OS=Escherichia phage vB_EcoM_AYO145A OX=1636202 GN=AYO145A_063 PE=4 SV=1 - [A0A0E3TAX3_9CAUD]	29,7869463	54,43	12	7	7	1,4957265	0,75675676	0,08464243	0,04282449	11	158	17,6711199	5,04638672		
A0A0A0RLD7	Uncharacterized protein OS=Escherichia phage vB_EcoM-VpaE1 OX=1555238 GN=VpaE1_040 PE=4 SV=1 - [A0A0A0RLD7_9CAUD]	28,8010304	43,09	3	5	5	1,06837607	0,54054054	0,07443868	0,03766195	9	123	14,3524314	8,88037109		
A0A384SJI0	Uncharacterized protein OS=Enterobacteria phage XTG1 OX=1651201 PE=4 SV=1 - [A0A384SJI0_9CAUD]	27,0090408	51	3	4	4	0,85470085	0,43243243	0,07695452	0,03893483	7	100	11,1065711	4,84326172		
O80234	Uncharacterized protein OS=Salmonella phage FelixO1 OX=77775 PE=4 SV=1 - [O80234_9CAUD]	18,0206006	44,95	19	4	4	0,85470085	0,43243243	0,07096138	0,03590262	5	109	12,0445926	10,081543		
A0A0A0RKH1	Uncharacterized protein OS=Escherichia phage vB_EcoM-VpaE1 OX=1555238 GN=VpaE1_053 PE=4 SV=1 - [A0A0A0RKH1_9CAUD]	14,701772	24,55	9	2	2	0,42735043	0,21621622	0,03660725	0,01852129	5	110	11,6739276	4,70361328		
A0A6B9WV3	HtjA OS=Escherichia phage orkinos OX=2696437 GN=orkinos_11 PE=4 SV=1 - [A0A6B9WV30_9CAUD]	11,4321929	25,44	5	3	3	0,64102564	0,32432432	0,03573192	0,01807842	4	169	17,9398616	5,33837891		
A0A0F6TJH5	Gp138_N domain-containing protein OS=Escherichia coli O157 typing phage 8 OX=1508683 GN=ECTP8_01530 PE=4 SV=1 - [A0A0F6TJH5_9C	8,45222759	12,08	4	2	2	0,42735043	0,21621622	0,01847354	0,00934661	4	207	23,1331056	5,04638672		
A0A6B9WRQ	Major capsid protein OS=Escherichia phage ityhuna OX=2696410 GN=ityhuna_34 PE=4 SV=1 - [A0A6B9WRQ2_9CAUD]	5,95321465	10,25	15	2	2	0,42735043	0,21621622	0,0119398	0,00604089	2	322	35,7920919	5,73193359		
A0A0A0RPQ	Uncharacterized protein OS=Escherichia phage vB_EcoM-VpaE1 OX=1555238 GN=VpaE1_047 PE=4 SV=1 - [A0A0A0RPQ7_9CAUD]	5,64408278	15,16	52	3	3	0,64102564	0,32432432	0,02506758	0,01268284	3	244	25,571904	4,61474609		
A0A2H4YE72	Baseplate J-like protein OS=Salmonella virus VSe102 OX=2053700 GN=vse102_068 PE=4 SV=1 - [A0A2H4YE72_9CAUD]	312,922321	66,26	2	2	23	0,42735043	2,48648649	0,00804877	0,0468308	95	489	53,0951105	4,61474609		
Q8XE41	Outer membrane porin C OS=Escherichia coli O157:H7 OX=83334 GN=ompC PE=3 SV=1 - [OMPC_ECO57]	16,7820916	17,98	1	6	6	1,28205128	0,64864865	0,03166852	0,01602256	6	367	40,4834642	4,77978516	**0,120198514**	0,05055621
P0C8Z2	Outer membrane protein A (Fragment) OS=Escherichia fergusonii OX=564 GN=ompA PE=3 SV=1 - [OMPA_ESCFE]	7,85221744	12,35	5	2	2	0,42735043	0,21621622	0,01635586	0,00827518	2	243	26,1282705	5,32568359		
P02938	Major outer membrane lipoprotein Lpp OS=Serratia marcescens OX=615 GN=lpp PE=3 SV=1 - [LPP_SERMA]	4,91874599	33,77	4	2	2	0,42735043	0,21621622	0,05189975	0,02625847	2	77	8,23415238	8,83642578		
P08267	Ferritin heavy chain OS=Gallus gallus OX=9031 GN=FTH PE=2 SV=2 - [FRIH_CHICK]	5,44234776	17,22	1	2	2	0,42735043	0,21621622	0,02027439	0,01025774	2	180	21,0783412	6,21435547		
P04264	Keratin, type II cytoskeletal 1 OS=Homo sapiens OX=9606 GN=KRT1 PE=1 SV=6 - [K2C1_HUMAN]	283,626522	55,28	1	30	34	6,41025641	3,67567568	0,09712656	0,0556929	82	644	65,999004	8,11865234	0,617537562	0,40633876
P13645	Keratin, type I cytoskeletal 10 OS=Homo sapiens OX=9606 GN=KRT10 PE=1 SV=6 - [K1C10_HUMAN]	174,70737	47,09	1	20	24	4,27350427	2,59459459	0,07268891	0,04413199	51	584	58,7916963	5,21142578		
P35908	Keratin, type II cytoskeletal 2 epidermal OS=Homo sapiens OX=9606 GN=KRT2 PE=1 SV=2 - [K22E_HUMAN]	163,247425	60,25	1	26	32	5,55555556	3,45945946	0,08495614	0,05290242	51	639	65,3932203	8,00146484		
P35527	Keratin, type I cytoskeletal 9 OS=Homo sapiens OX=9606 GN=KRT9 PE=1 SV=3 - [K1C9_HUMAN]	143,80245	61,8	1	25	26	5,34188034	2,81081081	0,08612211	0,04531606	45	623	62,0268176	5,23681641		
P02533	Keratin, type I cytoskeletal 14 OS=Homo sapiens OX=9606 GN=KRT14 PE=1 SV=4 - [K1C14_HUMAN]	60,1062043	29,66	1	4	13	0,85470085	1,40540541	0,01658667	0,02727386	20	472	51,5293865	5,16064453		
P13647	Keratin, type II cytoskeletal 5 OS=Homo sapiens OX=9606 GN=KRT5 PE=1 SV=3 - [K2C5_HUMAN]	54,5977678	29,83	1	11	18	2,35042735	1,94594595	0,03770337	0,03121506	21	590	62,33998	7,73779297		
P08779	Keratin, type I cytoskeletal 16 OS=Homo sapiens OX=9606 GN=KRT16 PE=1 SV=4 - [K1C16_HUMAN]	49,3896909	24,52	1	2	11	0,42735043	1,18918919	0,00834079	0,02320994	17	473	51,2362049	5,04638672		
P48668	Keratin, type II cytoskeletal 6C OS=Homo sapiens OX=9606 GN=KRT6C PE=1 SV=3 - [K2C6C_HUMAN]	34,3381491	19,68	1	3	11	0,64102564	1,18918919	0,01068584	0,01982368	14	564	59,9883084	8,00146484		
P02768	Albumin OS=Homo sapiens OX=9606 GN=ALB PE=1 SV=2 - [ALBU_HUMAN]	23,1588292	13,3	2	5	6	1,06837607	0,64864865	0,0154119	0,00935711	8	609	69,3214988	6,27783203		
O43790	Keratin, type II cuticular Hb6 OS=Homo sapiens OX=9606 GN=KRT86 PE=1 SV=1 - [KRT86_HUMAN]	21,5194813	12,76	2	7	7	1,4957265	0,75675676	0,02797513	0,0141539	9	486	53,4663013	5,65576172		
Q86YZ3	Hornerin OS=Homo sapiens OX=9606 GN=HRNR PE=1 SV=2 - [HORN_HUMAN]	19,6666114	8,07	1	6	6	1,28205128	0,64864865	0,00454261	0,00229832	7	2850	282,227637	10,0375977		
Q14525	Keratin, type I cuticular Ha3-II OS=Homo sapiens OX=9606 GN=KRT33B PE=1 SV=3 - [KT33B_HUMAN]	11,5209663	9,65	1	3	4	0,64102564	0,43243243	0,01387966	0,00936314	4	404	46,1845417	4,84326172		
Q5T749	Keratinocyte proline-rich protein OS=Homo sapiens OX=9606 GN=KPRP PE=1 SV=1 - [KPRP_HUMAN]	8,65619206	8,12	1	3	3	0,64102564	0,32432432	0,01000145	0,0050602	3	579	64,0932431	8,26513672		
Q5D862	Filaggrin-2 OS=Homo sapiens OX=9606 GN=FLG2 PE=1 SV=1 - [FILA2_HUMAN]	7,94559169	1,3	1	2	2	0,42735043	0,21621622	0,00172369	0,00087209	2	2391	247,927823	8,30908203		
P07477	Trypsin-1 OS=Homo sapiens OX=9606 GN=PRSS1 PE=1 SV=1 - [TRY1_HUMAN]	6,57659078	12,15	1	2	2	0,42735043	0,21621622	0,01610146	0,00814647	2	247	26,5410901	6,50732422		
P05109	Protein S100-A8 OS=Homo sapiens OX=9606 GN=S100A8 PE=1 SV=1 - [S10A8_HUMAN]	5,65728831	23,66	1	2	2	0,42735043	0,21621622	0,03946844	0,0199689	2	93	10,8276497	7,03466797		
P81605	Dermcidin OS=Homo sapiens OX=9606 GN=DCD PE=1 SV=2 - [DCD_HUMAN]	5,37954783	20	1	2	2	0,42735043	0,21621622	0,03789633	0,0191735	2	110	11,2768282	6,53662109		
P15924	Desmoplakin OS=Homo sapiens OX=9606 GN=DSP PE=1 SV=3 - [DESP_HUMAN]	4,78939581	0,59	1	2	2	0,42735043	0,21621622	0,00128887	0,0006521	2	2871	331,568719	6,81494141		
P00761	Trypsin OS=Sus scrofa OX=9823 PE=1 SV=1 - [TRYP_PIG]	91,7709126	25,11	1	4	4	0,85470085	0,43243243	0,03503762	0,01772714	30	231	24,3938063	7,18115234		
P00548	Pyruvate kinase PKM OS=Gallus gallus OX=9031 GN=PKM PE=2 SV=2 - [KPYM_CHICK]	55,9328496	48,87	1	18	18	3,84615385	1,94594595	0,06633841	0,03356365	20	530	57,9777786	7,60595703	**0,38496683**	0,2000583
P00940	Triosephosphate isomerase OS=Gallus gallus OX=9031 GN=TPI1 PE=1 SV=2 - [TPIS_CHICK]	49,8872182	72,18	1	13	13	2,77777778	1,40540541	0,10441289	0,05282728	17	248	26,6037821	7,19580078		
P02769	Albumin OS=Bos taurus OX=9913 GN=ALB PE=1 SV=4 - [ALBU_BOVIN]	26,5570006	14,33	1	6	7	1,28205128	0,75675676	0,01851379	0,01092814	9	607	69,2484438	6,17626953		
P51903	Phosphoglycerate kinase OS=Gallus gallus OX=9031 GN=PGK PE=2 SV=2 - [PGK_CHICK]	21,5115777	21,82	1	6	6	1,28205128	0,64864865	0,0286888	0,01451498	6	417	44,6882138	8,11865234		
P00565	Creatine kinase M-type OS=Gallus gallus OX=9031 GN=CKM PE=2 SV=1 - [KCRM_CHICK]	18,1870348	18,64	1	5	5	1,06837607	0,54054054	0,02467326	0,01248333	6	381	43,3009742	6,99072266		
P07322	Beta-enolase OS=Gallus gallus OX=9031 GN=ENO3 PE=1 SV=3 - [ENOB_CHICK]	17,0858734	15,67	1	5	5	1,06837607	0,54054054	0,02265123	0,0114603	6	434	47,1663525	7,60595703		
P00340	L-lactate dehydrogenase A chain OS=Gallus gallus OX=9031 GN=LDHA PE=1 SV=3 - [LDHA_CHICK]	15,5719421	17,17	1	5	5	1,06837607	0,54054054	0,02927763	0,0148129	5	332	36,4912034	7,89892578		
P00883	Fructose-bisphosphate aldolase A OS=Oryctolagus cuniculus OX=9986 GN=ALDOA PE=1 SV=2 - [ALDOA_RABIT]	14,9345086	20,05	5	5	5	1,06837607	0,54054054	0,02717251	0,01374782	6	364	39,3182672	8,08935547		
P93594	Beta-amylase Tri a 17 OS=Triticum aestivum OX=4565 GN=BMY1 PE=1 SV=1 - [AMYB_WHEAT]	13,837167	4,77	1	1	2	0,21367521	0,21621622	0,00377684	0,00382176	4	503	56,5750739	5,42724609		
P00356	Glyceraldehyde-3-phosphate dehydrogenase OS=Gallus gallus OX=9031 GN=GAPDH PE=2 SV=3 - [G3P_CHICK]	12,4989376	20,12	2	4	4	0,85470085	0,43243243	0,02395375	0,0121193	4	333	35,6812909	8,54345703		
P82993	Beta-amylase OS=Hordeum vulgare subsp. spontaneum OX=77009 GN=BMY1 PE=1 SV=1 - [AMYB_HORVS]	11,0319228	8,22	1	2	3	0,42735043	0,32432432	0,00717015	0,00544156	4	535	59,601338	6,01123047		
Q5ZLN1	Phosphoglycerate mutase 1 OS=Gallus gallus OX=9031 GN=PGAM1 PE=1 SV=3 - [PGAM1_CHICK]	9,56218171	17,72	1	3	3	0,64102564	0,32432432	0,02219629	0,01123012	3	254	28,8798501	7,48876953		
P10387	Glutenin, high molecular weight subunit DY10 OS=Triticum aestivum OX=4565 GN=GLU-D1-2B PE=3 SV=1 - [GLT0_WHEAT]	5,20731139	2,93	2	2	2	0,42735043	0,21621622	0,00614126	0,00310714	2	648	69,5867831	7,76708984		
					468	925										
